# STAR-GO: improving protein function prediction by learning to hierarchically integrate ontology-informed semantic embeddings

**DOI:** 10.1093/bioinformatics/btag146

**Published:** 2026-03-25

**Authors:** Mehmet Efe Akça, Gökçe Uludoğan, Arzucan Özgür, İnci M Baytaş

**Affiliations:** Department of Computer Engineering, Bogazici University, Bebek, Istanbul 34342, Turkiye; Department of Computer Engineering, Bogazici University, Bebek, Istanbul 34342, Turkiye; Department of Computer Engineering, Bogazici University, Bebek, Istanbul 34342, Turkiye; Department of Computer Engineering, Bogazici University, Bebek, Istanbul 34342, Turkiye

## Abstract

**Motivation:**

Accurate prediction of protein function is essential for elucidating molecular mechanisms and advancing biological and therapeutic discovery. Yet experimental annotation lags far behind the rapid growth of protein sequence data. Computational approaches address this gap by associating proteins with Gene Ontology (GO) terms, which encode functional knowledge through hierarchical relations and textual definitions. However, existing models often emphasize one modality over the other, limiting their ability to generalize, particularly to unseen or newly introduced GO terms that frequently arise as the ontology evolves, and making the previously trained models outdated.

**Results:**

We present STAR-GO, a Transformer-based framework that jointly models the semantic and structural characteristics of GO terms to enhance zero-shot protein function prediction. STAR-GO integrates textual definitions with ontology graph structure to learn unified GO representations, which are processed in hierarchical order to propagate information from general to specific terms. These representations are then aligned with protein sequence embeddings to capture sequence–function relationships. STAR-GO achieves state-of-the-art performance and superior zero-shot generalization, demonstrating the utility of integrating semantics and structure for robust and adaptable protein function prediction.

**Availability:**

Code and pre-trained models are available at https://github.com/boun-tabi-lifelu/stargo, https://doi.org/10.5281/zenodo.18643082

## Introduction

Proteins are essential macromolecules that perform diverse cellular functions, including catalyzing biochemical reactions, transmitting signals, and providing structural support. Understanding protein function, which is fundamental to biological research and therapeutic development, is a resource-intensive and time-consuming process. An extensive protein database, UniProt, contains over 190 million sequences of which only less than 1% have experimentally determined functional annotations (The UniProt Consortium 2021). Consequently, computational function prediction methods have become crucial for addressing this functional annotation gap.

The annotations of protein functions are standardized through Gene Ontology (GO) (Gene Ontology Consortium 2000, Gene Ontology Consortium 2023), a hierarchical classification scheme of three domains: molecular function (MF), biological process (BP), and cellular component (CC). GO contains a variety of information through its graph structure (e.g., ancestor-descendant relationships, part-of relationships, and regulation relationships), logical axioms, and textual descriptions (class labels and definitions). The rich GO structure presents both opportunities and challenges for computational approaches.

Protein function prediction is commonly posed as multi-label classification over the GO terms. While early studies transferred annotations from homologous proteins (Altschul et al. 1990), recent methods design deep function prediction models, integrating protein sequences (Kulmanov and Hoehndorf 2020, Cao and Shen 2021, Sanderson et al. 2023), structures (Gligorijević et al. 2021), their interactions (You et al. 2021), and literature (Yan et al. 2024). Despite their versatility, most deep protein function prediction models treat GO terms as independent labels, neglecting their directed acyclic graph (DAG) nature, which is characterized by rich axioms and curated term descriptions. However, these hierarchical and semantic features can be used to relate GO terms with proteins, thereby improving the generalization capability to novel functions, particularly in zero-shot prediction, where the goal is to infer functions linked to GO terms unseen during training or newly introduced in future ontology releases. Therefore, recent studies propose embedding methods that map ontology entities into continuous vector spaces, preserving their structure and relationships to incorporate GO characteristics into the learning problem.

General-purpose ontology embedding techniques such as OWL2Vec and EL Embeddings have been proposed and applied to GO. OWL2Vec integrates graph topology via random walks while combining logical axioms and lexical information (Chen et al. 2021), whereas EL Embeddings (Kulmanov et al. 2019) represent ontological relations geometrically in a continuous space. In addition, GO-specific ontology embedding techniques have also been introduced. anc2vec (Edera et al. 2022) is a GO-specific ontology embedding technique, where the ontological uniqueness, ancestor hierarchy, and sub-ontology memberships are embedded via an autoencoder setting. GT2Vec (Zhao et al. 2022) also employs an autoencoder to generate semantic GO embeddings by fine-tuning BioBERT (Lee et al. 2020) while preserving graph structure.

In line with advances in GO embedding techniques, protein function prediction models that incorporate GO embeddings into their architectures have also been recently proposed. DeepGOZero (Kulmanov and Hoehndorf 2022) and its successor DeepGO-SE (Kulmanov et al. 2024) employ EL Embeddings, representing GO terms as geometric objects (n-dimensional balls) constrained by logical axioms. This formulation encodes the ontology’s formal relationships, enabling zero-shot prediction for unseen GO terms. DeepGO-SE further introduces approximate semantic entailment by ensembling models built on pretrained protein language models (Rives et al. 2021), yet its representations remain fundamentally axiom-based. PFresGO (Pan et al. 2023) instead employs anc2vec (Edera et al. 2022) embeddings and integrates them with protein representations via cross-attention. While it captures hierarchical and membership relations, anc2Vec encodes GO terms as one-hot vectors, which limits its zero-shot capability. TransFew (Boadu and Cheng 2024) combines BioBERT-derived textual embeddings with GCN-encoded hierarchical relationships and fuses them with protein representations via cross-attention, targeting rare GO term prediction. Although these studies benefit from incorporating ontological structure into protein function prediction, none jointly optimizes semantic and structural GO embeddings within a hierarchical decoding framework that captures functional dependencies across ontology levels.

This study introduces STAR-GO, a Transformer-based framework that jointly embeds the structural and semantic characteristics of GO terms to enhance zero-shot protein function prediction. STAR-GO integrates hierarchical relations and textual definitions of GO terms, aligning ontology-informed embeddings with protein sequence representations to enable prediction of unseen functions. The framework has two main contributions: (i) GO term embeddings derived from a language model are refined via a structure-recovering autoencoder trained with multi-task supervision, preserving both semantic similarity and hierarchical dependencies for zero-shot inference without retraining; (ii) these enriched embeddings are incorporated into an encoder–decoder transformer, where GO terms are decoded in topological order using causal self-attention and linked to protein embeddings through cross-attention. The hierarchical decoding mechanism propagates information from general ancestors to specific child terms, capturing functional dependencies across levels. Evaluations under standard and zero-shot settings show that STAR-GO is competitive with state-of-the-art methods and demonstrates superior zero-shot generalization.

## Methods

### Dataset

We train and evaluate STAR-GO on a dataset curated from DeepFRI (Gligorijević et al. 2021), which is commonly used in the protein function prediction literature. The dataset comprises 36,641 protein sequences with coverage of 2,752 GO terms, including the following subontology terms: 489 Molecular Function, 1,943 Biological Process, and 320 Cellular Component, where each GO term is linked to at least 50 non-redundant Protein Data Bank (PDB) chains.

The dataset is split into training (80%; 29,902 sequences), validation (10%; 3,323 sequences), and test (10%; 3,416 sequences) sets (see Supplementary Table 1 for details, available as supplementary data at *Bioinformatics* online). The test set consists only of proteins with at least one experimentally validated functional annotation in each of the three GO categories, with protein chain lengths limited to 1,000 residues. To ensure non-redundancy between the training and test sets, cd-hit (Li and Godzik 2006) was applied using 95% sequence identity threshold.

For zero-shot evaluation, we follow DeepGOZero’s protocol (Kulmanov and Hoehndorf 2022), using the same similarity-based split of the UniProt/Swiss-Prot Knowledgebase (version 2021_04, on 29 September 2021; see Supplementary Table 2, available as supplementary data at *Bioinformatics* online).We hold out the same 16 GO terms as DeepGOZero (5 MF, 7 BP, 4 CC), which are selected from classes with >100 annotations. We remove all protein-class associations for these terms from the training set before applying the true path rule, which propagates annotations of GO terms to their ancestors, ensuring hierarchical consistency.

We use the basic version of GO with the true path rule applied for training and evaluations, specifically the June 2020 release ^1^ in the standard setting and the November 2021 release^2^ in the zero-shot setting.

### STAR-GO

STAR-GO is a Transformer-based architecture that integrates protein sequence representations with a novel GO embedding framework for protein function prediction. The GO embedder combines the semantic and structural information of GO terms by encoding term descriptions with their ontology relations. The sentence-level embeddings of GO terms, extracted using a sentence encoder, are mapped into the latent space of an autoencoder jointly trained for predicting the GO term’s aspect (i.e., MFO, BPO, CCO), its ancestor terms, and its identity. By jointly modeling semantic content and ontology hierarchy, this design enables zero-shot generalization to unseen GO terms.

**Figure 1:**
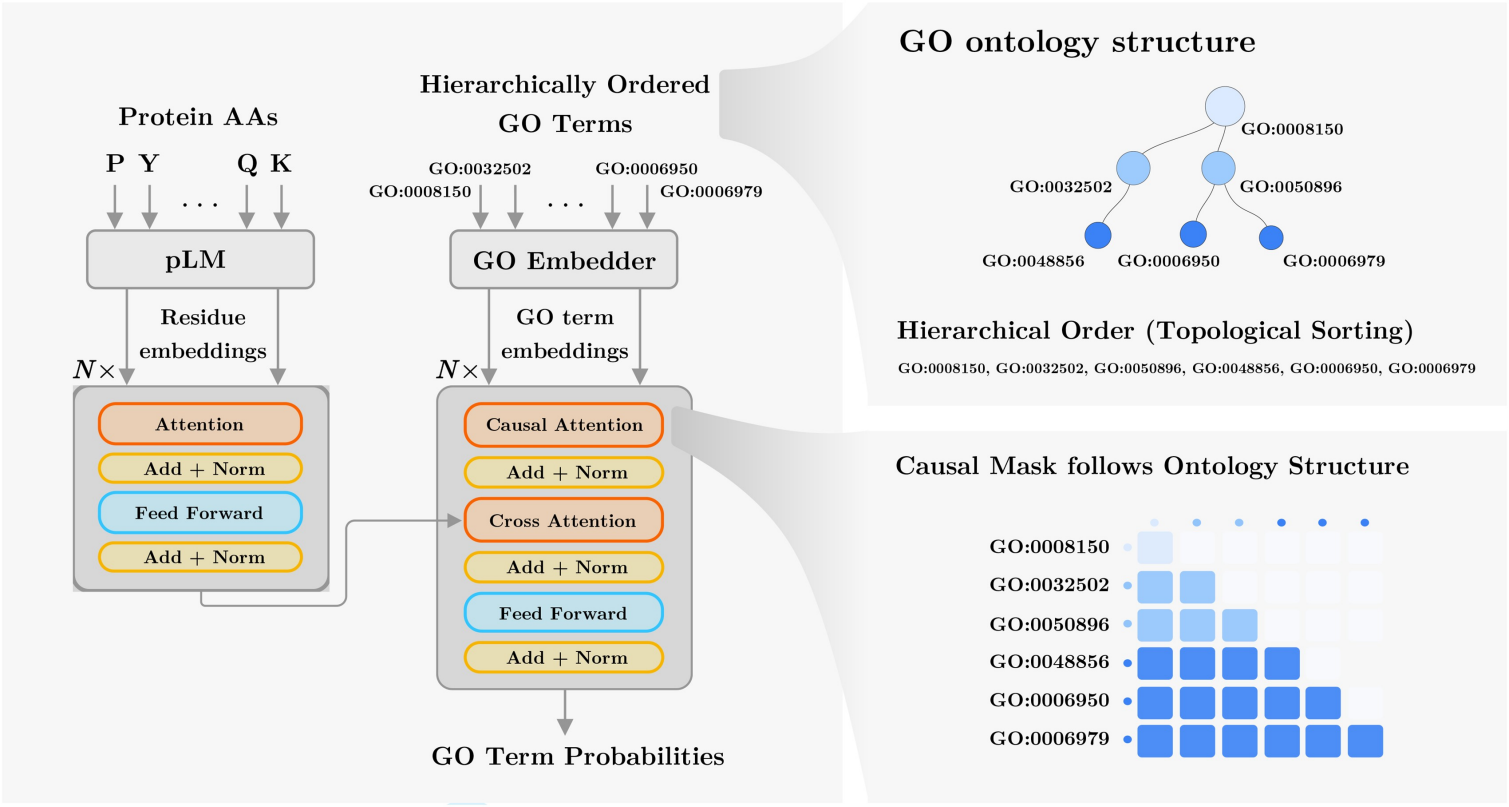
Overview of the proposed STAR-GO architecture. The GO embedding module derives sentence-level representations of term descriptions (e.g., using SBERT-BioBERT) and projects them into a latent space that reflects the ontology’s hierarchical structure. GO embeddings are hierarchically ordered, allowing the decoder to attend to ancestor terms and model dependencies across ontology levels.

STAR-GO adopts an encoder-decoder architecture, given in Fig. 1, where protein and GO embeddings are fused within a unified Transformer framework. The encoder takes protein embeddings obtained from a pretrained language model, while the decoder predicts GO term probabilities based on the fusion of the protein and GO representations via cross-attention. To ensure consistency with the hierarchical structure of the GO ontology, the decoder processes the GO embeddings arranged in a topological order from ancestors to descendants. Consequently, the proposed hierarchical decoding framework captures the dependencies across multiple functional levels.

### GO embeddings

GO is a structured vocabulary for protein functions, forming a directed acyclic graph (DAG), where nodes are terms and edges denote semantic relations (Gene Ontology Consortium 2000). The proposed embedding module aims to learn GO term embeddings by capturing the semantic meaning of term definitions and the structural relationships defined by the GO that are valuable for protein function prediction. We obtain sentence embeddings for each GO term definition using SBERT-BioBERT (Lee et al. 2020), pretrained with biomedical text. We apply mean pooling to token representations from its last layer to obtain GO term textual embeddings. The frozen embeddings serve as inputs to a trainable autoencoder that injects ontology structure via multi-task supervision (Edera et al. 2022). Unlike anc2Vec, our autoencoder learns the hierarchical structure of GO terms from their sentence embeddings, which encode the semantic similarity between the function descriptions, rather than relying on one-hot representations. The proposed encoder applies a linear projection, $z_{t}=W_{\text{emb}}x_{t}$, where $x_{t}\in R^{d_{t}}$ is the embedding of term *t*, and $W_{\text{emb}}\in R^{d_{z}\times d_{t}}$ is the projection matrix. The decoder learns to map $z_{t}$, ${\hat{y}}_{\text{anc}}=\phi (W_{\text{anc}}z_{t}+b_{\text{anc}}),{\hat{y}}_{\text{sub}}=\phi (W_{\;\text{sub}}z_{t}+b_{\text{sub}}),{\hat{y}}_{\text{id}}=\phi (W_{\text{id}}z_{t}+b_{\text{id}})$, using separate layers with softmax, ϕ(·), to recover the term’s ancestor set, its subontology, and its term identity with a combination of binary cross-entropy objectives:


(1) $$L_{\text{BCE}}=-\alpha_{\text{anc}}\sum_{i=1}^{N_{\text{terms}}}y_{\text{anc}}^{i}\;\text{log}\;\left({\hat{y}}_{\text{anc}}^{i}\right)$$

$$-\alpha_{\text{sub}}\sum_{i=1}^{3}y_{\text{sub}}^{i}\;\text{log}\;\left({\hat{y}}_{\text{sub}}^{i}\right)-\alpha_{\text{id}}\sum_{i=1}^{N_{\text{terms}}}x_{i}\;\text{log}\;\left({\hat{y}}_{\text{id}}^{i}\right)$$

Where $\alpha_{\text{anc}}$, $\alpha_{\text{sub}}$, and $\alpha_{\text{id}}$ are the loss weights balancing ancestor, subontology, and term ID loss terms, respectively, $N_{\text{terms}}$ is the number of terms, and $y_{\text{anc}}\in {\left\{0,1\right\}}^{N_{\text{terms}}}$ denotes the ground truth ancestor terms of x, and $y_{\text{sub}}\in {\left\{0,1\right\}}^{3}$ is the ground truth encoding of the subontology terms.

The anc2Vec (Edera et al. 2022) represents each GO term using one-hot vectors, where nonidentical terms are orthogonal and share limited similarity. In contrast, STAR-GO utilizes semantic embeddings of term descriptions, thereby it preserves relationships beyond the ontology graph. For example, Molecular Function terms GO : 0005524 (ATP binding) and GO : 0016887 (ATP hydrolysis activity) are functionally related since ATP hydrolysis inherently involves ATP binding. However, they share only the root ancestor molecular_function in the ontology, illustrating the gap between semantics and graph structure. The cosine similarity between the one-hot vectors of these terms is 0.48. With the anc2Vec embeddings, the similarity decreases to 0.29, resulting in a limited structural overlap. However, the similarities with our description-based embeddings are 0.69 and 0.68 before and after projection, respectively, indicating that semantic proximity is preserved. Finally, unlike anc2Vec, which assigns equal weights to each objective, we tune $\alpha_{\text{anc}},\alpha_{\text{sub}},\alpha_{\text{id}}$ to stabilize training and balance the influence of semantic and structural components.

### Transformer module

The proposed module learns to associate protein sequences with GO terms by jointly processing residue-level embeddings representing protein sequences and term-level embeddings representing GO semantics and hierarchy. The architecture follows an encoder-decoder design where cross-attention combines protein and GO representations. The encoder consists of $N_{\text{enc}}$ self-attention layers that contextualize residue embeddings, capturing dependencies along the sequence. The decoder comprises $N_{\text{dec}}$ layers that combine self-attention and cross-attention operations. The decoder’s self-attention operates over GO terms with a causal mask that respects their topological order, allowing information to flow from general to specific functions per the GO hierarchy. Cross-attention layers link the GO term representations with the residue embeddings, allowing each term to attend to the most informative residues relevant to its semantics. We adopt cross-attention as the fusion mechanism because it has been successfully applied in prior protein function prediction models, including PFresGO and TransFew, to integrate protein and GO term representations. The model is trained for the supervised function prediction task, encouraging the model to capture task-relevant associations between residue-level and GO-level representations. Our implementation builds on HuggingFace Transformers (Wolf *et al.* 2019) encoder and decoder layers. For a formal definition of the architecture, please see Supplementary Materials, available as supplementary data at *Bioinformatics* online.

#### Input and projection

Each protein sequence is represented as residue-level embeddings. We extract these embeddings from the final hidden layer of a pretrained protein language model, **ProtT5** (Elnaggar et al. 2022), which is state-of-the-art across diverse protein prediction tasks. For a sequence of length *L*, the residue embeddings, denoted as $X\in R^{L\times d_{\text{seq}}}$, are projected into the encoder’s latent space:


(2) $$H_{\text{enc}}^{\left(0\right)}=XW_{\text{seq}}+1b_{\text{seq}}^{⊤}\in R^{L\times d}.$$

#### Self-attention layers

The encoder refines the projected residue embeddings through $N_{\text{enc}}$ stacked self-attention layers, each comprising multi-head attention (Vaswani et al. 2017), a position-wise feed-forward network, and layer normalization with residual connections. After $N_{\text{enc}}$ layers, the encoder outputs refined residue embeddings $H_{\text{enc}}^{\left(N_{\text{enc}}\right)}$.

### Decoder

#### Input representation and ordering

Each GO term within a subontology is represented by the embeddings obtained from the GO embedding module, $E\in R^{T\times d_{\text{go}}}$, where *T* denotes the number of GO terms. To preserve the hierarchical dependencies among GO terms, they are arranged in a topological order $\left({\text{GO}}_{\pi (1)},\ldots ,{\text{GO}}_{\pi (T)}\right)$, where π defines a breadth-first traversal from root to leaves. This ordering enables information to propagate along parent-child relationships during decoding. The GO embeddings are then projected into the decoder’s latent space, producing the initial representation $H_{\text{dec}}^{\left(0\right)}$:


(3) $$H_{\text{dec}}^{\left(0\right)}=EW_{\text{go}}+1b_{\text{go}}^{⊤}\in R^{T\times d}.$$

#### Self-attention and cross-attention

The decoder consists of $N_{\text{dec}}$ layers, each containing causal self-attention, cross-attention, and a feed-forward sub-layer with residual connections and layer normalization. Causal self-attention is applied over GO terms using a lower-triangular mask ${\tilde{M}}_{\text{dec}}$, ensuring that each term attends only to terms preceding it in topological order, providing an inductive bias aligning with GO hierarchy. Subsequently, cross-attention incorporates the encoded protein representations $H_{\text{enc}}^{\left(N_{\text{enc}}\right)}$, allowing GO terms to attend to residue-level features relevant to their semantics. Thus, each GO term representation aggregates information from both its ancestors and the relevant residues of the protein.

### Prediction head and objective

The final decoder output is mapped through a two-layer feed-forward projection with GELU activation and sigmoid to get the predicted probability that the protein is associated with each GO term *t*. The model is trained using binary cross-entropy. During training, only the projection layers, Transformer blocks, and prediction head are optimized, while the pretrained protein and GO embedding modules remain frozen.

## Results

We compared STAR-GO with state-of-the-art models to evaluate its protein function prediction performance and generalization to unseen GO terms. We further examined the contribution of STAR-GO components with ablation studies. Performance was evaluated using $F_{\max}$ for protein-centric accuracy and Macro AUPR and AUC for term-centric generalization across GO terms. Implementation details, baselines, and evaluation metrics are provided in the Supplementary Materials, available as supplementary data at *Bioinformatics* online.

### STAR-GO achieves competitive performance

We compared STAR-GO with state-of-the-art baselines, including PFresGO, DeepFRI, TALE+, DeepGOZero, DeepGO-SE, and TransFew, using $F_{\text{max}}$, Macro AUPR, and AUC metrics (Table 1). Across all ontologies, STAR-GO exhibits competitive performance, matching or exceeding most baselines. In BP, DeepGO-SE attains the highest overall $F_{\text{max}}$ and Macro AUPR, while STAR-GO achieves the best AUC (0.989). Within CC ontology, STAR-GO surpasses all baselines in Macro AUPR and AUC while maintaining comparable $F_{\text{max}}$ to PFresGO. For MF, STAR-GO performs on par with DeepGO-SE, achieving close $F_{\text{max}}$ and AUC values. STAR-GO consistently ranks among the top-performing methods and achieves the strongest AUC values across all ontologies, indicating strong term-level discriminability.

**Table 1:** Comparison of STAR-GO with state-of-the-art. STAR-GO performs competitively across all ontologies.

Ontology	Method	$F_{\text{max}}$	Macro AUPR	AUC
**BP**	PFresGO	0.568	0.293	0.839
	DeepFRI	0.540	0.261	0.858
	TALE+	0.554	0.302	0.811
	DeepGOZero	0.565	0.294	0.768
	DeepGO-SE	**0.574**	**0.325**	0.968
	TransFew	0.559	0.254	0.847
	STAR-GO	0.548	0.288	**0.989**
**CC**	PFresGO	**0.674**	0.361	0.884
	DeepFRI	0.613	0.274	0.884
	TALE+	0.610	0.325	0.849
	DeepGOZero	0.534	0.315	0.738
	DeepGO-SE	0.638	0.352	0.974
	TransFew	0.650	0.347	0.890
	STAR-GO	0.659	**0.379**	**0.988**
**MF**	PFresGO	0.691	0.602	0.924
	DeepFRI	0.625	0.494	0.915
	TALE+	0.662	0.564	0.884
	DeepGOZero	0.719	0.614	0.893
	DeepGO-SE	**0.722**	**0.648**	0.991
	TransFew	0.695	0.593	0.946
	STAR-GO	0.719	0.620	**0.995**

Notably, it obtains the highest AUC scores, demonstrating its term-level discriminability. The highest values across subontologies and metrics are shown in **bold.**

### STAR-GO generalizes to unseen GO terms

For zero-shot evaluation, we compared STAR-GO with DeepGOZero, DeepGO-SE, and TransFew, which have zero-shot capabilities, using the zero-shot dataset, where selected GO terms were excluded from all protein–term associations. To assess the contributions of different GO information sources, we ablated the structural and textual components of our GO embedding module, denoted STAR${\;}_{S}$ (structural only, using anc2vec embeddings) and STAR${\;}_{T}$ (textual-only, using SBERT-BioBERT embeddings), and STAR${\;}_{ST}$ model combining both representations. All models, including baselines, were trained on the zero-shot dataset and evaluated on the full test set, with term-specific AUCs reported for held-out GO terms in Table 2.

**Table 2:** Zero-shot performance of STAR-GO with unseen GO term AUCs.

		Zero-shot						Supervised		
Ontology	Term	STAR${\;}_{ST}$	STAR${\;}_{T}$	STAR${\;}_{S}$	DeepGOZero	DeepGO-SE	TransFew	STAR${\;}_{ST}$	DeepGOZero	DeepGO-SE
MF	GO : 0001227	0.891	**0.944**	0.557	0.257	0.759	0.395	**0.955**	0.932	0.952
	GO : 0001228	0.938	**0.949**	0.508	0.574	0.791	0.533	**0.961**	0.948	**0.961**
	GO : 0003735	**0.923**	0.915	0.301	0.400	0.066	0.459	0.994	0.940	**0.995**
	GO : 0004867	0.742	0.884	0.807	**0.972**	0.568	0.175	0.955	0.985	**0.992**
	GO : 0005096	0.904	**0.907**	0.750	0.847	0.517	0.359	**0.962**	0.938	0.735
BP	GO : 0000381	0.973	**0.988**	0.957	0.855	0.751	0.423	0.987	0.906	**0.992**
	GO : 0032729	0.895	0.895	0.948	0.870	**0.982**	0.866	0.893	0.932	**0.996**
	GO : 0032755	0.895	0.889	0.957	0.719	**0.966**	0.754	0.939	0.884	**0.979**
	GO : 0032760	0.900	0.883	**0.930**	0.861	0.803	0.691	**0.950**	0.925	0.821
	GO : 0046330	0.949	**0.960**	0.923	0.855	0.866	0.496	**0.966**	0.904	0.888
	GO : 0051897	0.893	**0.942**	0.913	0.772	0.929	0.683	**0.962**	0.888	0.957
	GO : 0120162	0.734	0.779	**0.811**	0.637	0.491	0.600	0.817	0.738	**0.957**
CC	GO : 0005762	**0.998**	**0.998**	0.991	0.889	0.600	0.090	**0.999**	0.874	0.995
	GO : 0022625	**0.989**	0.937	0.950	0.898	0.487	0.397	**0.995**	0.893	0.991
	GO : 0042788	0.940	**0.963**	0.930	0.858	0.532	0.520	**0.990**	0.889	**0.990**
	GO : 1904813	0.675	0.806	**0.903**	0.653	0.658	0.724	0.895	0.792	**0.955**

The zero-shot columns denote that models were optimized on an ablated dataset excluding the reported GO terms from all protein–term associations. Supervised columns reports the results for the corresponding protein-term pairs included during training. STAR variants use different GO embeddings: *S* (structural, anc2Vec), *T* (textual, SBERT-BioBERT), and *ST* (combined, proposed in this work). The best AUC within each section is shown in **bold**.

Across the 16 held-out GO terms, STAR-GO variants collectively achieve the highest zero-shot AUCs in 13 cases, consistently outperforming DeepGOZero, DeepGO-SE and TransFew. Performance varies by ontology and GO embeddings: STAR${\;}_{T}$ achieves the best results on most Molecular Function and Biological Process terms (e.g., GO : 0001228 = 0.949, GO : 0046330 = 0.960), highlighting the strength of semantic information captured from textual definitions. STAR${\;}_{S}$ performs best on a few terms (e.g., GO : 0032760 = 0.930, GO : 1904813 = 0.903) but performs poorly for MF terms. The combined STAR${\;}_{ST}$ model maintains stable and competitive performance across all ontologies, closely matching the textual variant.

An independent subsumption-prediction evaluation of the GO embeddings further contextualized these results (see Supplementary Table S3, available as supplementary data at *Bioinformatics* online). The anc2vec performed the best in recovering hierarchical parent–child relations, confirming its strong topological representation of GO. However, anc2vec embeddings generalized less effectively to unseen terms, particularly for MF terms, resulting in the lower zero-shot AUCs of STAR${\;}_{S}$. Conversely, SBERT-BioBERT embeddings performed worse in subsumption prediction but generalized better to unseen terms, indicating that semantic representations transfer more effectively to novel ontology concepts. The integrated STAR${\;}_{ST}$ model reflects this complementarity, achieving balanced performance across ontologies by leveraging both semantic and structural information. While STAR${\;}_{S}$ requires retraining when new GO terms are added due to the fixed anc2vec embeddings, STAR${\;}_{T}$ and STAR${\;}_{ST}$ can generate embeddings for novel terms directly from textual definitions, enabling continuous adaptation to ontology updates without retraining. A seed sensitivity analysis over five random seeds confirmed the stability of these zero-shot results, with low standard deviations across all held-out terms (see Supplementary Tables S5 and S6, available as supplementary data at *Bioinformatics* online).

### Hierarchical ordering and ontology-informed embeddings improve performance

We ablated STAR-GO to quantify the contributions of GO embedding design, integration architecture, and hierarchical ordering of GO terms. Table 3 shows that the best performance was achieved by the decoder-based model using our integrated GO embedding module with hierarchically ordered GO terms. This variant shows strength in Macro AUPR scores, achieving 0.288, 0.379, and 0.620 for BP, CC, and MF, respectively, and achieves the highest $F_{\text{max}}$ score (0.719) for MF subontology.

**Table 3:** Ablation study of STAR-GO across different ontologies and three aspects: (1) GO embeddings: None (learned from scratch), Structural (anc2vec), Textual (SBERT-BioBERT), or Combined (proposed integration of structural and textual embeddings); (2) Architecture: decoder with causal attention, encoder with bidirectional attention, or MLP using concatenated embeddings of the mean-pooled protein embeddings and GO embeddings; (3) GO term ordering: whether terms are hierarchically ordered in the decoder (✓).

Model Configurations			Macro AUPR			Micro AUPR			AUC			Fmax		
GO Embeddings	Architecture	GO Ordering	BP	CC	MF	BP	CC	MF	BP	CC	MF	BP	CC	MF
None	Decoder	√	0.230	0.378	0.583	0.315	0.457	0.634	0.985	0.988	0.992	0.548	0.658	0.691
Structural	Decoder	√	0.267	0.366	0.608	0.322	0.449	0.660	0.983	0.989	0.994	**0.553**	0.653	0.700
Textual	Decoder	√	0.195	0.296	0.388	0.250	0.344	0.346	0.975	0.980	0.981	0.438	0.575	0.393
Combined	MLP	x	0.277	0.351	0.567	0.343	0.453	0.622	**0.990**	0.992	**0.995**	0.534	0.662	0.654
Combined	Encoder	x	–	0.340	0.577	–	**0.463**	0.633	–	**0.990**	**0.995**	–	**0.667**	0.670
Combined	Decoder	x	0.281	0.361	0.610	0.340	0.443	0.650	0.985	0.989	0.993	0.542	0.647	0.707
Combined	Decoder	√	**0.288**	**0.379**	**0.620**	**0.351**	0.455	**0.675**	0.989	0.988	**0.995**	0.548	0.659	**0.719**

Results are shown for Biological Process (BP), Cellular Component (CC), and Molecular Function (MF) predictions using multiple metrics. (-) indicates experiments not completed due to GPU memory constraints. The last configuration corresponds to the proposed model, STAR-GO. The best scores within each section is shown in **bold.**

Removing either the textual or structural component from the GO embeddings results in performance degradation, most notably for BP, where Macro AUPR drops from 0.288 to 0.267 (structural-only) or 0.195 (textual-only). The textual-only variant performed substantially worse here than in the zero-shot setting, where it achieved the highest AUCs. This discrepancy suggests that textual embeddings (SBERT-BioBERT) are effective for transferring to unseen terms but less so when trained end-to-end, whereas structural embeddings (anc2vec) provide a stronger inductive bias and stability when full supervision is available. STAR-GO performed the best across ontologies leveraging textual and structural information.

Replacing hierarchical ordering with a flat ordering results in an overall decrease in performance, confirming that the hierarchical ordering contributes a useful inductive bias by enforcing the GO structure. Training GO embeddings from scratch (the “None” configuration in Table 3, without any structural or textual pretrained GO embeddings) performs significantly worse, demonstrating the importance of pretrained GO embeddings for generalization.

We further compared different strategies for integrating GO embeddings with protein representations. In the decoder-based configuration, GO embeddings attend to each other with a causal mask such that GO terms are processed in hierarchical or GO ID-based order. The encoder-based configuration, which attends GO term sequence embeddings bidirectionally without ordered decoding, achieved competitive AUCs in CC and MF (0.990 and 0.995) and the highest CC $F_{\text{max}}$ (0.667), but could not be evaluated on BP due to memory constraints. The MLP integration, which pairs mean-pooled protein embeddings with individual GO term embeddings, produced similarly high AUCs (up to 0.995) but underperformed in $F_{\text{max}}$, Macro AUPR, and Micro AUPR compared to the decoder variant with GO hierarchical ordering. Additionally, we evaluated ESM-1 and ESM-2 protein embeddings and found that ProtT5 performed better for our model (see Supplementary Table S4, available as supplementary data at *Bioinformatics* online).

### STAR-GO discovers key residues in the zero-shot setting

The decoder cross-attention weights indicate protein residues the model considers relevant to each GO term. To aggregate the signals across heads and layers, we applied recursive averaging to cross-attention matrices, inspired by attention rollout (Abnar and Zuidema 2020). Let ${\bar{A}}^{\left(l\right)}=\frac{1}{H}\sum_{h=1}^{H}A_{h}^{\left(l\right)}\in R^{T\times L}$ denote the head-averaged cross-attention at decoder layer *l*. The rollout is computed recursively as:


(4) $$\begin{array}{c} R^{\left(1\right)}={\bar{A}}^{\left(1\right)}\\ R^{\left(l\right)}=\frac{1}{2}\left({\bar{A}}^{\left(l\right)}+R^{\left(l-1\right)}\right),\;l=2,\ldots ,N_{\text{dec}}, \end{array}$$

Equally weighting the current layer’s attention and the accumulated score. Row *t* of the final rollout $R=R^{\left(N_{\text{dec}}\right)}\in R^{T\times L}$ yields the per-residue attention profile $r_{t}\in R^{L}$ for GO term *t*. We applied this procedure to STAR-GO in the zero-shot setting to determine whether cross-attention captures biologically meaningful signals for a function the model has never seen. We selected two held-out Molecular Function terms related to DNA-binding transcription factor activity: GO : 0001228 (*transcription activator*, zero-shot AUC = 0.938) and GO : 0001227 (*transcription repressor*, zero-shot AUC = 0.891). We computed attention rollout scores for three PDB chains: (GO : 0001228) 4AWL chain B (NF-YB), 2F8X chain C (RBPJ/CSL), and (GO : 0001227) 2HDC chain A (FOXD3). As ground truth for DNA-binding residues, we obtained experimentally determined DNA-contact sites from the BioLiP database (Yang et al. 2013).

**Figure 2:**
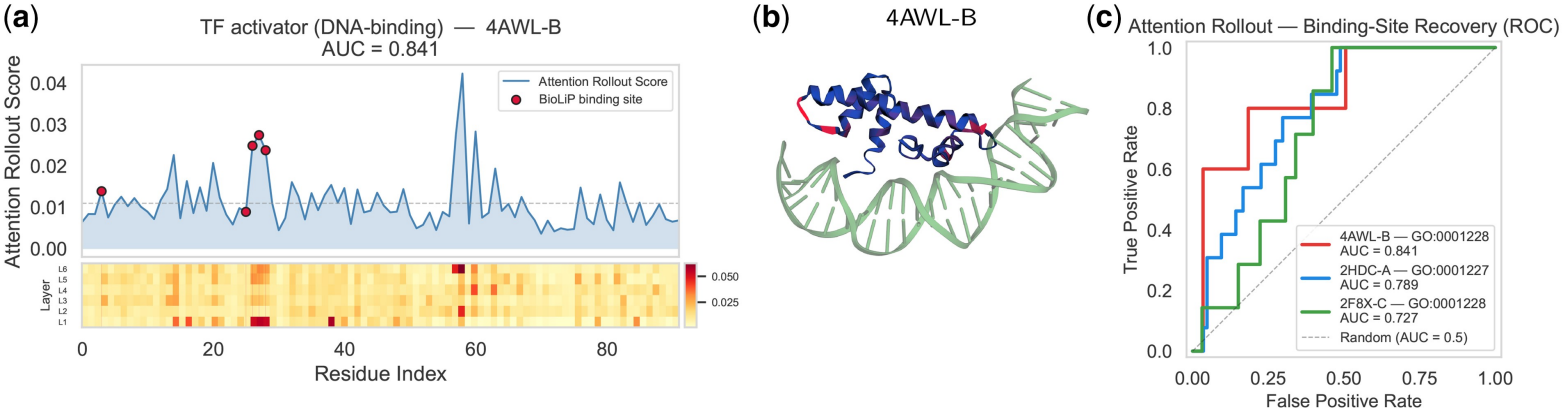
Interpretability analysis of STAR-GO’s zero-shot cross-attention for two held-out DNA-binding transcription factor terms: GO : 0001228 (activator) and GO : 0001227 (repressor). **(a)** Attention rollout and binding-site residues for 4AWL-B. Above: per-residue attention rollout scores with DNA-contact sites (red). Below: heatmap of per-residue attention averaged over heads for each decoder layer. **(b)** 3-D structure of 4AWL-B coloured by attention rollout score (blue → red indicates increasing attention), with DNA shown in green. **(c)** ROC curves evaluating agreement between attention-derived residue scores and BioLiP binding-site annotations.

Fig. 2a shows the per-residue attention rollout profile for 4AWL-B, with head-averaged attention at each decoder layer as a heatmap and the aggregated rollout score plotted above. The BioLiP-annotated DNA-contact residues (red dots) coincide with regions of elevated attention, particularly around residues 25–30, corresponding to the NF-YB DNA-contact region, with a strong signal in the early decoder layers. The 3D structure (Fig. 2b) confirms that residues receiving high rollout scores (red) are spatially proximal to the bound DNA (green). Fig. 2c reports ROC curves for all three chains: 4AWL-B achieves an AUROC of 0.841, 2HDC-A 0.789, and 2F8X-C 0.727, all substantially above the random baseline.

Critically, both GO : 0001228 and GO : 0001227 were excluded from all protein-term associations during training; STAR-GO has never observed any protein annotated with either term. STAR-GO’s elevated attention to experimentally validated DNA-contact residues arises solely from the information in the term’s embedding and its alignment with sequence features learned through supervision on other GO terms. This demonstrates that the decoder learns a generalizable mapping between GO term semantics and residue-level features that extends beyond the training vocabulary.

## Discussion

This work demonstrates that integrating semantic and structural information yields a richer representation of GO terms for protein function prediction. The proposed GO embedding module, integrated within an encoder-decoder framework and combined with hierarchical decoding, enhances zero-shot prediction performance while maintaining competitive protein-centric and improved term-centric results. Moreover, because our GO embedding method requires only term descriptions at inference, it can be applied across GO releases without retraining.

The ablation results provide additional insight into the interaction between semantic and structural information. The combined model does not consistently outperform the single-modality variants. Both the text-only and structure-only models achieve higher AUCs for certain terms, suggesting that each information source captures complementary yet occasionally conflicting aspects of functional similarity. This variability indicates that the current fusion mechanism may sometimes overemphasize one modality, depending on the characteristics of individual terms. Future work will develop adaptive fusion strategies that dynamically balance semantic and structural contributions, improving robustness across GO terms. Our implementation employs specific pretrained language models for both protein and GO embeddings. However, the framework can incorporate alternative protein representations or additional modalities, such as structure-based or protein–protein interaction embeddings. Future work will also explore the fusion of complementary representations to further enhance the performance.

## Conclusion

We presented STAR-GO, a Transformer-based framework that jointly embeds the semantic and structural characteristics of GO terms to enhance zero-shot protein function prediction. The proposed GO embedding module refines language-model-derived representations through structural regularization, preserving both textual semantics and hierarchical relationships of GO terms. This design enables compatibility with evolving GO releases without retraining, while hierarchical decoding provides an inductive bias for propagating information from general to specific terms. Our results demonstrate that STAR-GO achieves strong zero-shot generalization and competitive performance compared to state-of-the-art methods. Our findings highlight that semantic and structural representations capture complementary aspects of functional similarity: textual embeddings provide adaptability and compatibility across GO releases, while structural information encodes relational context within the ontology. Future work will focus on developing adaptive fusion mechanisms and extending STAR-GO to include additional modalities for both proteins and GO terms. This study represents an important step toward generalizable function prediction models that are resilient to the continual evolution of biological ontologies.

## Supplementary Material

btag146_Supplementary_Data

## Data Availability

We publicly release our code at https://github.com/boun-tabi-lifelu/stargo, which has also been archived together with model checkpoints on Zenodo (https://zenodo.org/records/18643081). We use datasets from the PFresGO and DeepGOZero studies and provide scripts to download these datasets.

## Footnotes

1
https://release.geneontology.org/2020-06-01/ontology/go-basic.obo

2
https://release.geneontology.org/2021-11-16/ontology/go-basic.obo
