# Supplementary material for: STAR-GO: improving protein function prediction by learning to hierarchically integrate ontology-informed semantic embeddings
Source: Bioinformatics. 2026 Mar 25;42(4):btag146. doi: 10.1093/bioinformatics/btag146 (PMC13120692; doi:10.1093/bioinformatics/btag146)
Supplement: btag146_Supplementary_Data [file btag146_supplementary_data.pdf]

SUPPLEMENTARY MATERIALS:
STAR-GO: IMPROVING PROTEIN FUNCTION PREDICTION BY LEARNING TO HIERARCHICALLY INTEGRATE ONTOLOGY-INFORMED SEMANTIC EMBEDDINGS

Dataset statistics

Table S1 summarizes the number of protein sequences and annotated GO terms in the training, validation, and test splits used for model development. Table S2 presents the corresponding statistics for the dataset employed in zero-shot evaluation, including per-subontology protein and term counts.

Table S1: Dataset statistics for training, validation and test splits. The number of protein sequences in each split and the number of GO terms with annotations per ontology.

Subontology	Train	Test	Validation	Num. terms
MF	29,902	3,416	3,323	489
BP	29,902	3,416	3,323	1,943
CC	29,902	3,416	3,323	320

Table S2: Statistics for UniProtKB–SwissProt 2021_4 dataset used in zero-shot evaluation. Per-subontology protein counts in each split and term count is shown.

Subontology	Terms	Training	Validation	Testing
MF	6,868	34,716	3,851	4,712
BP	21,381	47,733	5,552	5,444
CC	2,832	48,318	4,970	5,969

GO Embedding Module

Figure 1 summarizes the GO embedding framework described in Section *GO Embeddings*, highlighting the integration of semantic (text-based) and structural (ontology-based) information via a projection autoencoder trained with multi-task objectives.

Transformer Module

The encoder and decoder of STAR-GO follow the standard pre-norm Transformer architecture [Vaswani et al., 2017] with multi-head attention (MHA), position-wise feed-forward networks (FFN), and layer normalization (LN). Below we provide the full equations for completeness.

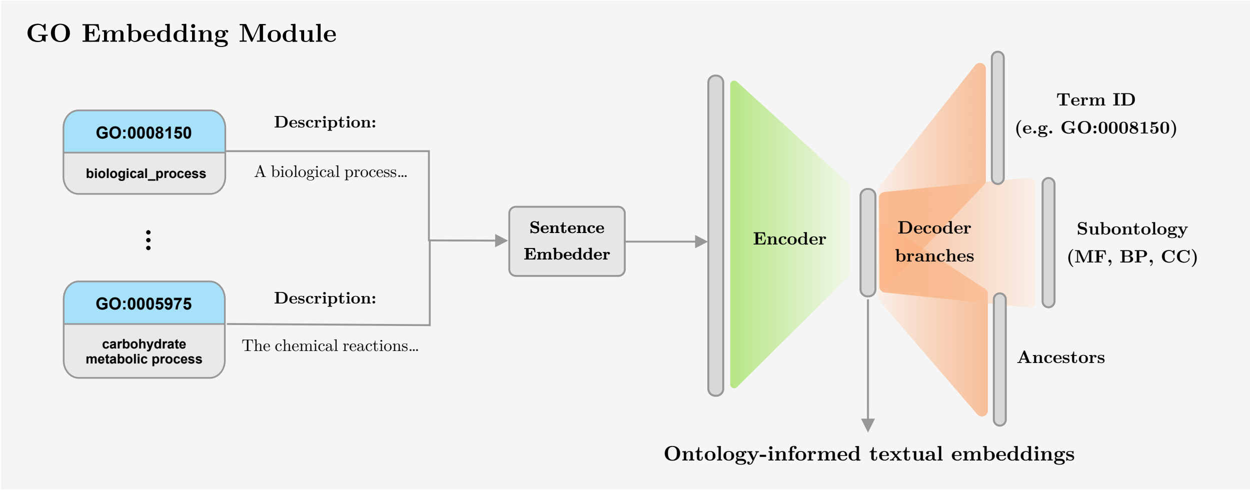

Figure 1: Overview of the GO embedding module. Sentence embeddings obtained from SBERT-BioBERT are projected into a latent space through a trainable autoencoder. The encoder maps GO term definitions into a semantic-structural representation, while three decoder branches jointly reconstruct (i) ancestor terms, (ii) subontology, and (iii) term identity. Multi-task supervision aligns the semantic embeddings of related GO terms with their hierarchical relationships in the ontology.

Encoder Self-Attention

Let $\mathbf{H}_{\text{enc}}^{(l)} \in \mathbb{R}^{L\times d}$ denote the hidden representation of the protein sequence at the l-th encoder layer, where $\mathbf{H}_{\text{enc}}^{(0)}$ is the projected input (see main text). Each layer updates these representations as follows:

$$\hat{\mathbf{H}}_{\text{enc}}^{(l)} = \text{LN}\left(\mathbf{H}_{\text{enc}}^{(l-1)} + \text{MHA}\left(\mathbf{H}_{\text{enc}}^{(l-1)}, \mathbf{H}_{\text{enc}}^{(l-1)}; \tilde{\mathbf{M}}_{\text{seq}}\right)\right), \tag{1}$$

$$\mathbf{H}_{\text{enc}}^{(l)} = \text{LN}\left(\hat{\mathbf{H}}_{\text{enc}}^{(l)} + \text{FFN}\left(\hat{\mathbf{H}}_{\text{enc}}^{(l)}\right)\right) \tag{2}$$

where MHA denotes multi-head attention, FFN is a position-wise feed-forward network, LN denotes layer normalization, and $\tilde{\mathbf{M}}_{\text{seq}}$ is an additive mask for sequence padding. After N_{enc} layers, the encoder outputs refined residue embeddings $\mathbf{H}_{\text{enc}}^{(N_{\text{enc}})}$.

Decoder Self-Attention and Cross-Attention

Let $\mathbf{H}_{\text{dec}}^{(l)} \in \mathbb{R}^{T\times d}$ denote the hidden representation of the GO terms at decoder layer l, where $\mathbf{H}_{\text{dec}}^{(0)}$ is the projected GO embedding input (see main text). Each decoder layer is computed as:

$$\textbf{Self-attention: } \hat{\mathbf{H}}_{\text{dec}}^{(l)} = \text{MHA}\left(\mathbf{H}_{\text{dec}}^{(l-1)}, \mathbf{H}_{\text{dec}}^{(l-1)}; \tilde{\mathbf{M}}_{\text{dec}}\right), \tag{3}$$

$$\mathbf{S}_{\text{dec}}^{(l)} = \text{LN}\left(\mathbf{H}_{\text{dec}}^{(l-1)} + \hat{\mathbf{H}}_{\text{dec}}^{(l)}\right), \tag{4}$$

$$\textbf{Cross-attention: } \tilde{\mathbf{H}}_{\text{dec}}^{(l)} = \text{MHA}\left(\mathbf{S}_{\text{dec}}^{(l)}, \mathbf{H}_{\text{enc}}^{(N_{\text{enc}})}; \mathbf{0}\right), \tag{5}$$

$$\mathbf{C}_{\text{dec}}^{(l)} = \text{LN}\left(\mathbf{S}_{\text{dec}}^{(l)} + \tilde{\mathbf{H}}_{\text{dec}}^{(l)}\right), \tag{6}$$

$$\textbf{Feed-forward: } \mathbf{H}_{\text{dec}}^{(l)} = \text{LN}\left(\mathbf{C}_{\text{dec}}^{(l)} + \text{FFN}\left(\mathbf{C}_{\text{dec}}^{(l)}\right)\right). \tag{7}$$

The decoder's self-attention mask $\tilde{\mathbf{M}}_{\text{dec}}$ is a lower-triangular matrix that respects the topological ordering of GO terms, ensuring that each term attends only to its ancestors and preceding terms in the hierarchy. Cross-attention uses no mask ($\mathbf{0}$), allowing each GO term to attend to all encoded protein residues.

Prediction Head and Training Objective

For each GO term t, the final decoder output is mapped through a two-layer feed-forward projection:

$$\mathbf{u}_t = \text{GELU}\Big(\mathbf{H}_{\text{dec}}^{(N_{\text{dec}})}[t,:]\mathbf{W}_1 + \mathbf{b}_1\Big), \tag{8}$$

$$z_t = \mathbf{u}_t\mathbf{W}_2 + b_2, \quad \hat{y}_t = \sigma(z_t) \in (0,1). \tag{9}$$

where $\hat{y}_t$ denotes the predicted probability that the protein is associated with GO term t, and $\sigma(\cdot)$ is the sigmoid activation function.

Given binary ground-truth $\mathbf{y} \in \{0,1\}^T$, where $y_t = 1$ if the protein is annotated with GO term t and 0 otherwise, the model is trained using binary cross-entropy:

$$\mathcal{L}_{\text{BCE}} = \frac{1}{T}\sum_{t=1}^{T}\Big(\log(1+\exp(z_t)) - y_t z_t\Big). \tag{10}$$

Evaluation Metrics

We evaluate performance using the standard metrics of the Critical Assessment of Functional Annotation (CAFA) challenges[Zhou et al., 2019] including macro-averaged Area Under the Precision-Recall Curve (AUPR), F_{max} score, and micro-averaged Area Under the ROC Curve (AUC). Before evaluation, all predictions and labels are propagated up the Gene Ontology hierarchy according to the true path rule.

- **Protein-centric $\mathbf{F_{max}}$:** This is the protein-centric maximum for the F_1 measure. For a threshold t, define precision $P_t = \frac{TP_t}{TP_t+FP_t}$ and recall $R_t = \frac{TP_t}{TP_t+FN_t}$, where TP_t, FP_t, and FN_t represents true positives, false positives, and false negatives at threshold t. The F-measure at threshold t is calculated as $F_t = \frac{2\cdot P_t\cdot R_t}{P_t+R_t}$. F_{max} is defined as:
$$F_{max} = \max_{t\in[0,1]} F_t \tag{11}$$
When evaluating protein function prediction, the protein-centric $\mathbf{F_{max}}$ accounts for the GO term hierarchy by propagating predictions along the ontology structure.
- **Macro AUPR:** For each GO term g in the set of all terms G, an individual AUPR value is calculated as:
$$\text{AUPR}_g = \int_0^1 P_g(r)dr \tag{12}$$
where $P_g(r)$ is the precision at recall level r for term g. The Macro AUPR is then:
$$\text{Macro AUPR} = \frac{1}{|G|}\sum_{g\in G}\text{AUPR}_g \tag{13}$$
This gives equal weight to each term regardless of its prevalence in the dataset.
- **AUC (Area Under the ROC Curve):** For ROC calculation, the true positive rate TPR $= \frac{TP}{TP+FN}$ is plotted against the false positive rate FPR $= \frac{FP}{FP+TN}$ at various thresholds. The micro-averaged AUC is calculated by first flattening all predictions and true labels across all protein-term pairs into a single vector, then calculating
$$\text{AUC} = \int_0^1 \text{TPR}(\text{FPR}^{-1}(f))\,df \tag{14}$$
where $\text{FPR}^{-1}(f)$ is the threshold that gives an FPR of f.

The metrics above, calculated as defined in CAFA guidelines, provide a comprehensive view of model performance, covering both protein-level accuracy (F_{max}, AUC) and term-specific prediction quality (Macro AUPR).

Baselines

We evaluated STAR-GO against five baseline methods selected for their methodological relevance, zero-shot capabilities, or state-of-the-art performance.

Methods with similar architectures. PFresGO [Pan et al., 2023] combines ProtT5 protein representations with anc2vec GO embeddings through cross-attention in an auto-encoder framework with non-feedforward residual attention blocks. Unlike STAR-GO 's encoder-decoder architecture with semantic GO representations, PFresGO relies solely on graph-derived hierarchical information. TALE [Cao and Shen, 2021] employs a Transformer encoder to learn joint protein-GO embeddings, using convolution with softmaxed similarity scores for prediction.

Zero-shot capable methods. DeepGOZero [Kulmanov and Hoehndorf, 2022] pioneered zero-shot protein function prediction by representing GO terms as geometric n-balls that satisfy formal Description Logic axioms, operating on InterPro domain features rather than sequence embeddings. DeepGO-SE [Kulmanov et al., 2024] extends this geometric approach through approximate semantic entailment across multiple models while incorporating ESM2 protein language models. More recently, TransFew [Boadu and Cheng, 2024] follows a method similar to STAR-GO by combining BioBert embeddings of GO definitions with GO hierarchy through a GCN, and cross-attends them with refined ESM embeddings.

Structure-based. DeepFRI [Gligorijević et al., 2021] is a structure-based function predictor, using graph convolutional networks on residue contact maps to capture spatial functional determinants.

Implementation Details

The models were trained and evaluated using the PyTorch 2.5.1 [Paszke et al., 2017], HuggingFace Transformers 4.47.0 [Wolf et al., 2019], and PyTorch Lightning 2.4.0[Falcon and The PyTorch Lightning team, 2019] libraries. Our model implementation utilized HuggingFace's BERT encoder and decoder layers, replacing the trained embeddings module with the frozen embeddings from our method. For the encoder variant, we utilized a fully enabled attention mask in the self-attention layer, effectively making it an encoder.

Experiment tracking and hyperparameter tuning were performed on the Weights&Biases platform [Biewald, 2020]. Hyperparameter tuning was utilized to optimize the model's performance and experiments were performed with multiple variants of the model. In particular, we experimented with the fixed ordering of Gene Ontology terms in the decoder input, testing the variations of: ordered, unordered GO terms with a causal mask and unordered GO terms without a causal mask. We also evaluated an ablation variant in which protein and GO term embeddings were concatenated and passed through an MLP prediction head. We utilized PyTorch Lightning's Learning Rate Finder to find the optimal learning rate for each model architecture.

For the GO embedding module fine-tuning, we adapted the Anc2vec training procedure to incorporate our semantic embeddings using a multi-task loss formulation with an ExponentialDecay learning rate scheduler. The embedding dimension was set to $d = 200$. Training proceeded for 100 epochs with batch size 32, using scheduler parameters `initial_lr=0.001`, `decay_rate=0.9`, and `decay_steps=10000`. Loss weights were set to $\alpha_{\text{anc}} = 1.0$, $\alpha_{\text{sub}} = 0.5$, and $\alpha_{\text{id}} = 0.3$ for the ancestor prediction, subontology classification, and term identity reconstruction tasks, respectively.

For STAR-GO , we use a hidden size $d = 256$ in all Transformer blocks with 6 encoder and 6 decoder layers, each with 8 attention heads and a feed-forward intermediate dimension of 1024. Dropout is 0.1 on hidden activations and 0.1 on attention probabilities; activations use GELU and we set `layer_norm_eps` to $1!\times!10^{-12}$. GO embeddings are 200-d for Anc2Vec and our learned GO embeddings, and 768-d when using the SBERT-BioBERT textual encoder; protein residues are projected to the shared hidden size d We train with AdamW (learning rate 6×10^{-5} selected by a PyTorch Lightning learning-rate finder, weight decay 0.01), without a learning-rate scheduler, for up to 100 epochs with early stopping (patience 10) on minimum validation loss. To standardize compute, we employ gradient accumulation together with Fully Sharded Data Parallel training, yielding an effective batch size of 32 across runs. Mixed precision uses `bf16` (`bf16-mixed` in PyTorch).

We adopted PFresGO's official evaluation code[1] for metric computation. All baseline results are taken from the PFresGO study, except for DeepGO-SE, which we retrained and evaluated for consistency. For zero-shot experiments, both DeepGOZero and DeepGO-SE were retrained under our ablation protocol.

Subsumption prediction with GO embeddings

We compared our GO embedding module against several baseline GO embedding techniques on the subsumption prediction task (Table S3), including OWL2Vec* [Chen et al., 2021], anc2Vec [Edera et al., 2022], and GT2Vec [Zhao et al., 2022]. To assess the contribution of semantic information, we also evaluated pretrained language models that encode GO term definitions without structural supervision. Specifically, we included two task-specific BERT models:

[1] `https://github.com/BioColLab/PFresGO`

BioBERT [Lee et al., 2020], pretrained on large-scale biomedical text, and *SBERT-BioBERT* [Reimers and Gurevych, 2019], which fine-tunes BioBERT on sentence similarity tasks using the SentenceTransformers framework.

The evaluation was performed using the GO subsumption dataset and evaluation metrics of OWL2Vec* [Chen et al., 2021]. The dataset is a split of all GO subsumption triples, i.e., `is_a`, `part_of` relations between GO terms. We evaluated the `go-basic` and go editions of the GO. The basic edition contains *part of*, *is a*, *regulates*, and *negatively regulates*. The full edition go additionally contains *has part* and *occurs in*. It is generally considered unsafe to propagate annotations with the complete edition since the additional relations introduce cycles to the graph [Gene Ontology Consortium, 2023].

For each embedding technique, we trained multiple models to predict the likelihood of a subsumption relationship between any two GO terms and selected the best-performing one. This model was then used for a ranking task: for each child term in the test set, we ranked all other GO terms as potential parents based on the predicted likelihood. We evaluated these rankings using standard information retrieval metrics. Hits@k measures the percentage of times a true parent is found within the top k ranked candidates; we report for k=1, 5, and 10. Mean Reciprocal Rank (MRR) evaluates the average inverse rank of the first correct parent, providing a single score for overall ranking quality. Higher values for all metrics indicate better performance.

As shown in Table S3, anc2vec achieved the highest accuracy in recovering subsumption relations, consistent with its explicit optimization for structural hierarchy. Sentence-based embeddings performed worse overall, though fine-tuned semantic embeddings (SBERT-BioBERT) provided a substantial improvement over non-fine-tuned BioBERT, demonstrating the effectiveness of fine-tuning the SBERT-BioBERT embeddings. Our GO embedding module, which integrates semantic embeddings with structural reconstruction objectives, performed comparably to OWL2Vec* across metrics and surpassed purely semantic models. Despite anc2vec's stronger subsumption scores, our ablation study (main text, Table 3) showed that these embeddings generalized less effectively to downstream protein function prediction. In contrast, our module's semantic–structural integration improved transfer to function prediction, confirming the value of including semantic information alongside hierarchical structure.

Table S3: Performance of GO embedding techniques. anc2vec and OWL2Vec* are the same as in their respective papers. BioBERT uses mean-pooled sentence embeddings of term descriptions. SBERT-BioBERT is a BioBERT model fine-tuned for sentence similarity tasks. Our GO embedding method fine-tunes SBERT-BioBERT further with structure recovery objectives. We evaluated on two editions of GO: full and basic.

GO Edition	Method	Hits@1	Hits@10	Hits@5	MRR
go	anc2vec	**0.077**	**0.365**	**0.245**	**0.170**
	Ours	0.063	0.333	0.220	0.149
	OWL2Vec*	0.071	0.343	0.230	0.158
	SBERT-BioBERT	0.060	0.297	0.190	0.137
	BioBERT	0.008	0.112	0.058	0.047
go-basic	anc2vec	**0.092**	**0.414**	**0.290**	**0.196**
	Ours	0.069	0.340	0.233	0.157
	OWL2Vec*	0.066	0.333	0.221	0.152
	SBERT-BioBERT	0.054	0.292	0.188	0.131
	BioBERT	0.022	0.156	0.097	0.071
	GT2Vec	0.006	0.067	0.028	0.038

Impact of residue embeddings across different protein language models

To assess the impact of the protein language model on downstream function prediction, we compared three residue-level embedding methods within STAR-GO's architecture: ESM-1b [Rives et al., 2021], ESM-2 [Lin et al., 2022], and ProtT5 [Elnaggar et al., 2022]. All other model components, including the GO embedding module, decoder architecture, and training procedure, were held constant; only the frozen residue embeddings provided to the encoder were varied. Table S4 reports results under our standard supervised evaluation protocol across all three ontologies.

ProtT5 embeddings consistently outperform both ESM variants across most metrics. The improvement is most pronounced for Molecular Function, where ProtT5 achieves an $F_{\max}$ of 0.719 compared to 0.675 for both ESM-1b and ESM-2, and a Macro AUPR of 0.620 versus 0.574–0.576. Similar gains are observed for Biological Process and Cellular Component in Macro AUPR and Micro AUPR. AUC scores are comparable across all three embeddings, indicating that the discriminative advantage of ProtT5 is most evident in precision-recall–based metrics where ranking

quality among positive terms matters most. These results motivated our adoption of ProtT5 as the default protein encoder in STAR-GO.

Table S4: Comparison of protein language model embeddings for protein function prediction. We evaluate three residue-level embedding methods: ESM1b, ESM2, and ProtT5. Results are reported across Biological Process (BP), Cellular Component (CC), and Molecular Function (MF) ontologies. ProtT5 embeddings (used in STAR-GO) consistently outperform ESM variants across most metrics, with particularly notable gains in Macro AUPR and Fmax.

Embeddings	Macro AUPR			Micro AUPR			AUC			Fmax		
Residue Embeddings	BP	CC	MF	BP	CC	MF	BP	CC	MF	BP	CC	MF
ESM1b	0.253	0.329	0.574	0.321	0.408	0.631	0.980	**0.988**	**0.995**	0.539	0.639	0.675
ESM2	0.268	0.349	0.576	0.321	0.406	0.628	0.975	0.985	0.994	0.544	0.640	0.675
ProtT5	**0.288**	**0.379**	**0.620**	**0.351**	**0.455**	**0.675**	**0.989**	**0.988**	**0.995**	**0.548**	**0.659**	**0.719**

Zero-shot experiment sensitivity analysis

To assess the stability of STAR-GO's zero-shot predictions with respect to random initialization, we repeated the zero-shot evaluation across five independent training seeds, keeping all hyperparameters fixed. Tables S5 and S6 report per-term AUC scores for each seed along with the mean and standard deviation.

Across all 16 held-out GO terms, the model exhibits low variance in most terms, with most standard deviations below 0.03. Cellular Component terms show particularly stable predictions, exemplified by GO:0005762 (mean AUC $= 0.9961 \pm 0.0016$). Overall, the consistently low standard deviations confirm that the zero-shot generalization is robust and not an artifact of a particular random seed.

Table S5: Zero-shot protein function prediction AUC scores (Biological Process) across 5 seeds.

	Biological Process						
Seed	GO:0000381	GO:0032729	GO:0032755	GO:0032760	GO:0046330	GO:0051897	GO:0120162
1	0.9735	0.8936	0.9042	0.9064	0.9685	0.9073	0.7754
2	0.9831	0.9201	0.9152	0.9630	0.9006	0.9034	0.8103
3	0.9709	0.8891	0.9030	0.9254	0.9609	0.9347	0.8290
4	0.9715	0.9058	0.9070	0.8935	0.9281	0.9223	0.8051
5	0.9849	0.9106	0.9162	0.9132	0.9556	0.9341	0.8115
Mean $\pm$ Std	0.9768 ± 0.0067	0.9038 ± 0.0126	0.9091 ± 0.0062	0.9203 ± 0.0265	0.9427 ± 0.0281	0.9203 ± 0.0146	0.8063 ± 0.0195

Table S6: Zero-shot protein function prediction AUC scores (Cellular Component & Molecular Function) across 5 seeds.

	Cellular Component				Molecular Function				
Seed	GO:0005762	GO:0022625	GO:0042788	GO:1904813	GO:0001227	GO:0001228	GO:0003735	GO:0004867	GO:0005096
1	0.9941	0.9672	0.8809	0.7675	0.8956	0.9271	0.7650	0.6246	0.9306
2	0.9948	0.9216	0.9056	0.7231	0.9270	0.9476	0.9219	0.8552	0.9367
3	0.9973	0.9746	0.9625	0.7587	0.9476	0.9526	0.6933	0.9030	0.9243
4	0.9975	0.9622	0.9254	0.6598	0.9387	0.9413	0.8459	0.7896	0.9405
5	0.9969	0.8599	0.9041	0.7939	0.9253	0.9442	0.9375	0.7253	0.9184
Mean $\pm$ Std	0.9961 ± 0.0016	0.9371 ± 0.0478	0.9157 ± 0.0305	0.7406 ± 0.0518	0.9269 ± 0.0197	0.9426 ± 0.0096	0.8327 ± 0.1039	0.7796 ± 0.1096	0.9301 ± 0.0090

References

L. Biewald. Experiment tracking with weights and biases, 2020. URL `https://www.wandb.com/`.

F. Boadu and J. Cheng. Improving protein function prediction by learning and integrating representations of protein sequences and function labels. *Bioinform. Adv.*, 4(1):vbae120, 2024. doi: 10.1093/bioadv/vbae120.

Y. Cao and Y. Shen. TALE: Transformer-based protein function annotation with joint sequence–label embedding. *Bioinformatics*, 37(18):2825–33, 2021. doi: 10.1093/bioinformatics/btab198.

J. Chen et al. OWL2Vec*: Embedding of OWL ontologies. *Mach. Learn.*, 110, 2021. doi: 10.1007/s10994-021-05997-6.

A. A. Edera, D. H. Milone, and G. Stegmayer. Anc2vec: Embedding gene ontology terms by preserving ancestors relationships. *Brief. Bioinform.*, 23(2):bbac003, 2022. doi: 10.1093/bib/bbac003.

A. Elnaggar, M. Heinzinger, C. Dallago, and et al. ProtTrans: Towards cracking the language of lifes code through self-supervised deep learning and high performance computing. *IEEE Trans. Pattern Anal. Mach. Intell.*, 2022. doi: 10.1109/TPAMI.2021.3095381.

W. Falcon and The PyTorch Lightning team. PyTorch Lightning, 2019.

Gene Ontology Consortium. The gene ontology knowledgebase in 2023. *Genetics*, 224(1):iyad031, 2023. doi: 10.1093/genetics/iyad031.

V. Gligorijević et al. Structure-based protein function prediction using graph convolutional networks. *Nat. Commun.*, 12(1):3168, 2021. doi: 10.1038/s41467-021-23303-9.

M. Kulmanov and R. Hoehndorf. DeepGOZero: improving protein function prediction from sequence and zero-shot learning based on ontology axioms. *Bioinformatics*, 38(Supplement_1):i238–45, 2022. doi: 10.1093/bioinformatics/btac256.

M. Kulmanov et al. Protein function prediction as approximate semantic entailment. *Nat. Mach. Intell.*, 6(2):220–8, 2024. doi: 10.1038/s42256-024-00795-w.

J. Lee et al. BioBERT: A pre-trained biomedical language representation model for biomedical text mining. *Bioinformatics*, 36(4):1234–40, 2020. doi: 10.1093/bioinformatics/btz682.

Z. Lin et al. Evolutionary-scale prediction of atomic level protein structure with a language model, 2022.

T. Pan et al. PFresGO: An attention mechanism-based deep-learning approach for protein annotation by integrating gene ontology inter-relationships. *Bioinformatics*, 39(3):btad094, 2023. doi: 10.1093/bioinformatics/btad094.

A. Paszke et al. Automatic differentiation in PyTorch. In *NIPS-W*, 2017.

N. Reimers and I. Gurevych. Sentence-BERT: Sentence embeddings using siamese BERT-networks, 2019.

A. Rives et al. Biological structure and function emerge from scaling unsupervised learning to 250 million protein sequences. *Proc. Natl. Acad. Sci. U.S.A.*, 2021. doi: 10.1073/pnas.2016239118.

A. Vaswani et al. Attention is all you need, 2017.

T. Wolf et al. Huggingface's transformers: State-of-the-art natural language processing. *CoRR*, abs/1910.03771, 2019.

L. Zhao et al. Learning representations for gene ontology terms by jointly encoding graph structure and textual node descriptors. *Brief. Bioinform.*, 23(5):bbac318, 2022. doi: 10.1093/bib/bbac318.

N. Zhou, Y. Jiang, T. R. Bergquist, A. J. Lee, B. Z. Kacsoh, A. W. Crocker, K. A. Lewis, G. Georghiou, H. N. Nguyen, M. N. Hamid, et al. The CAFA challenge reports improved protein function prediction and new functional annotations for hundreds of genes through experimental screens. *Genome Biol.*, 20(1):244, 2019.
